# Accumulating evidence across studies: Consistent methods protect against false findings produced by p-hacking

**DOI:** 10.1371/journal.pone.0307999

**Published:** 2024-08-29

**Authors:** Duane T. Wegener, Jolynn Pek, Leandre R. Fabrigar

**Affiliations:** 1 Department of Psychology, Ohio State University, Columbus, Ohio, United States of America; 2 Department of Psychology, Queen’s University, Kingston, Ontario, Canada; Curtin University of Malaysia, MALAYSIA

## Abstract

Much empirical science involves evaluating alternative explanations for the obtained data. For example, given certain assumptions underlying a statistical test, a “significant” result generally refers to implausibility of a null (zero) effect in the population producing the obtained study data. However, methodological work on various versions of p-hacking (i.e., using different analysis strategies until a “significant” result is produced) questions whether significant p-values might often reflect false findings. Indeed, initial simulations of single studies showed that the potential for finding “significant” but false findings might be much higher than the nominal .05 value when various analysis flexibilities are undertaken. In many settings, however, research articles report multiple studies using consistent methods across the studies, where those consistent methods would constrain the flexibilities used to produce high false-finding rates for simulations of single studies. Thus, we conducted simulations of study sets. These simulations show that consistent methods across studies (i.e., consistent in terms of which measures are analyzed, which conditions are included, and whether and how covariates are included) dramatically reduce the potential for flexible research practices (p-hacking) to produce consistent sets of significant results across studies. For p-hacking to produce even modest probabilities of a consistent set of studies would require (a) a large amount of selectivity in study reporting and (b) severe (and quite intentional) versions of p-hacking. With no more than modest selective reporting and with consistent methods across studies, p-hacking does not provide a plausible explanation for consistent empirical results across studies, especially as the size of the reported study set increases. In addition, the simulations show that p-hacking can produce high rates of false findings for single studies with very large samples. In contrast, a series of methodologically-consistent studies (even with much smaller samples) is much less vulnerable to the forms of p-hacking examined in the simulations.

## Introduction

Potential problems with replication of empirical research results have received much attention over the last 10–15 years. Lack of replication has been attributed to low levels of statistical power in original research [1–3]. Others point to various practices representing “researcher degrees of freedom” (RDFs) [4] or “questionable research practices” (QRPs), such as analyzing only subsets of measures based on results of those analyses, analyzing with and without various covariates, or adding data until a result becomes significant [cf. 5–8] (Manapat and colleagues [9] provide a taxonomy of RDFs and QRPs). Manapat and colleagues use the QRP terminology to refer to situations in which people undertake such practices as a way to enhance the significance of their results (aka p-hacking) [10] or when they use a method known to have subpar performance [9]. Regardless of the term(s) used, the concern with RDFs, QRPs, or p-hacking has been that their use might be associated with “false findings” (i.e., statistically significant findings that occur despite null effects in the population; cf. Type I errors). Because null population effects would tend to produce non-significant results in future research, such false findings would thus be associated with replication failures. That is, if a given practice, such as flexibly analyzing subsets of measures [11], falsely identifies “findings” when the population effect is null, any such “finding” is unlikely to be replicated in later research.

### Significance testing: Evaluating consistency with a null model

Let us start by considering where in the research evaluation process such issues arise. Hypothesis testing is typically used to evaluate how plausible it would be to argue that the observed data come from a population null effect with sampling variation (i.e., a null generating model). In such contexts, given that assumptions of the statistical test are satisfied, significant results suggest that the obtained data are quite inconsistent with the null model. Several recent criticisms have suggested, however, that statistical significance can be misleading because the findings are false (i.e., occurring despite population null effects). Criticisms based on statistical power have suggested that a large portion of significant results can be false when the prior probability of true null effects is high and statistical power is low [3]. Also, criticisms related to p-hacking have suggested that the significant tests themselves might occur with much higher than the nominal probability even with null effects in the population (e.g., over 60% rather than the nominal 5%) [4]. In these cases, then, traditional assumptions of the meaning of significant results have been questioned.

### P-hacking in single-study versus multi-study contexts

Initial treatments of p-hacking focused on the simplest case of single research studies or (especially for discussions of statistical power) treated all studies as independent and exchangeable when characterizing the literature more broadly [12, 13]. In such settings, many of the illustrations given and conclusions made have appeared rather dire. For example, estimates of false finding rates in a literature consisting of studies with power of .2 and a prior probability of a true null hypothesis of .9 would have a false finding rate of .69 [1]. That is, of all the studies rejecting the null hypothesis in that literature, 69% of the rejections would be “false” (i.e., rejection of a true null hypothesis). Similarly, in a summary of work on p-hacking Nelson and colleagues [13] noted that,

“In ‘False-Positive Psychology’ (Simmons et al. 2011), we demonstrated that p-hacking represents a major threat to the validity of all empirical research that relies on hypothesis testing…. It is now clear that, with enough analytic flexibility, *p*-hacking can turn any false hypothesis into one that has statistically significant support” (p. 516).

Yet, the psychological literature is not generally characterized by articles reporting single one-off independent studies. Rather, articles often report multiple studies and/or a program of research consisting of a series of studies that build on one another (often quite directly). For example, in a review of articles published in the *Journal of Personality and Social Psychology* in 2014, Flake and colleagues [14] reported an average of 4.02 studies per article (*SD* = 2.16). Similarly, despite a much more limited length of articles, in a review of every empirical article published in *Psychological Science* in 2017 and 2021, Hoisington-Shaw and colleagues [15] reported an average of 2.38 (*SD* = 1.72) and 2.48 (*SD* = 2.26) studies within an article, respectively. Thus, in many cases, evidence supporting or opposing a particular claim takes into account evidence across a (sometimes sizable) set of studies [16, 17].

What, then, are the implications of statistical power or p-hacking for evidence accumulated across sets of studies? Wegener and colleagues [13] addressed the topic of statistical power. One way to consider the impact of power is to take a Bayesian approach and examine how data beyond a first study might change beliefs about the existence (or lack thereof) of a studied pattern in the population. The same mathematical formulas that underlie claims of high potential for false findings when considering independent single studies suggest that additional studies producing directionally consistent (and at least sometimes significant) results should foster greater confidence that the population effect is not null. Another way to consider accumulation of evidence across studies is to examine the strength and consistency of results across the entire set of available studies. This could be done in various ways including examination of the proportion of studies from a set reaching significance [13, 18], the proportion of studies whose results fall in a particular direction (e.g., using a Sign Test) [19], or meta-analyzing the set of studies [17, 18, 21]. Examining the full set of studies highlights that evidence can be highly inconsistent with a null population effect even when not all studies individually reach statistical significance [13, 16–18, 20, 21]. Thus, consideration of study sets might lead one to evaluate evidence somewhat differently than one might when focusing on single studies. When focusing on single studies, a set of 12 studies in which only 4 or 5 reach significance might seem quite mixed and uncertain. Yet, when compared against the likely profile of studies implied by a null population, 4 or 5 significant studies falling in the same direction would be highly unlikely [13].

Unfortunately, previous discussions of study sets have not considered the possibility of p-hacking. If, as claimed by Nelson and colleagues [22], any study can be turned into a significant result, perhaps 4 or 5 (or even more) of the 12 studies could occur simply because the researchers were selectively employing different analysis approaches for the different studies in ways that made them look stronger than they really were. Indeed, Nelson and colleagues [22] noted that,

“It is tempting to take comfort in the fact that psychology publications usually contain more than one experiment (see, e.g., Stroebe 2016, section 1.6.1). Even if a single study’s false-positive rate is as high as 61%, the odds of getting four false positives for a single article (in the predicted direction) is nevertheless quite low: (0.61/2)^4^ = 0.8%. However, this framing of the problem assumes two things—one that is never true and one that is sometimes not true. The first assumption is that researchers publish every study. They do not. If a researcher is willing to file-drawer even a small number of studies, a false-positive four-study article becomes easy enough to produce [Pashler & Harris (2012, argument 2, p. 533) provide an excellent discussion of this issue].… The other assumption is that 61% represents some sort of upper bound, when, in fact, that estimate is likely to be conservative, particularly if researchers are able to flexibly contort the hypotheses to fit the data that they observe (Kerr 1998). In truth, it is not that hard to get a study’s false-positive rate to be very close to 100%, in which case even a multistudy article’s false-positive rate will be close to 100%.”

Dire indeed. It would seem quite problematic if p-hacking easily takes a study’s false-positive rate to be close to 100%. Perhaps even more so, it would be highly problematic if that would translate into a multi-study article’s false finding rate being close to 100%. However, such claims of disaster also make a number of assumptions, some of which likely make the claims more dire than might be reflected in everyday research. We address such assumptions in the following section of the paper.

### Assumptions underlying concerns about p-hacking and examination of whether or how such assumptions apply to multi-study contexts

#### Arbitrary direction of results

One key assumption that any discussion of single studies avoids altogether is the direction of results. That is, single-study false finding rates are generally given as if any direction of effect is fine. Sometimes researchers might accept any direction of effect as long as the test is significant. After all, it might be possible to generate explanations for any direction after the fact [23]. Thus, direction for an initial study is sometimes quasi-arbitrary but perhaps not nearly as much as implied by the single-study discussions.

There are two aspects to consider. Sometimes a particular direction of effect is implausible because of the logic of a causal variable. For instance, in the persuasion domain, making weak/specious arguments more persuasive than strong/compelling arguments (assuming no confounds) is quite difficult [24]. Given the nature of the causal variable, one can easily explain situations when one finds no difference between strong and weak arguments (e.g., when lacking ability to process [25]), or one can explain results in which strong arguments are more persuasive than weak arguments (e.g., when motivation and ability are both high [26]). Yet, it is much harder to explain why message recipients would be more persuaded by arguments that are weak and specious than by arguments that are strong and compelling. A second related constraint is the broader literature. For totally novel effects that have no broader context, an arbitrary direction of an effect might often be acceptable. For established literatures in which a finding must fit with other findings, however, it gets much harder, and a researcher will face skepticism and probably need to clear a higher empirical hurdle to convince other researchers of the novel effect. For example, setting aside the conceptual nature of argument quality, there is now an established literature with many (perhaps hundreds) of studies producing either null effects of argument quality (e.g., when distraction is high or involvement is low [25, 26]) or strong arguments being more persuasive than weak arguments (e.g., when distraction is low or involvement is high [25, 26]). A new researcher suggesting that a particular condition leads to more persuasion by weak arguments than by strong arguments, especially a condition that seems conceptually similar to the conditions that have led to null or positive effects in many previous studies, would receive much skepticism and require more data (likely a number of studies) to convince readers. Thus, it seems that effect direction is often not as arbitrary as single-study discussions of false findings might imply.

However, especially when developing a research program that tests a particular prediction or theory, one direction of effect is strongly preferred over the other on theoretical grounds. Even when one does not start with a clear prediction, when conducting a set of studies, the direction of an initial result sets an expectation for future studies. If the results of those studies are directionally inconsistent and roughly balanced across directions (as would be expected with a population null effect), most researchers would (or should) be uncomfortable publishing either the set as a whole or either “side” of the set (because in both cases the population direction remains uncertain). Moreover, in such cases, presentation of the whole set would be met with skepticism, and meta-analyses or integrative data analyses would show that the effect across all the studies is null. Also, those attempting to replicate or build on a selective publication of one side of the distribution would have much difficulty reproducing the original pattern, thereby calling the original “result” (a false finding) into question.

We maintain that it is, in fact, difficult to approach anything close to a 100% false finding rate when only one particular direction of effect is acceptable. As addressed later in this article, when consistency in direction is required of the studies with significant results along with consistency in the methods used, it becomes quite difficult to maintain a particular direction of effect using datasets in which the population effects are null even with somewhat extreme versions of flexibility in the analyses.

#### Hiddenness of flexibilities

When claiming dramatic influences of p-hacking, other key assumptions include that reviewers and editors would find any significant result to be compelling regardless of the methods used or, perhaps, that the relevant methods remain unknown to research consumers. To create false finding rates near 100% (for individual studies or, especially, for multi-study sets) would likely require quite an array of RDFs/QRPs (or very severe versions). Yet, many such practices should be quite apparent to research consumers. For example, if some studies in a set include covariates and others do not, if the nature of the covariates change from study to study, or if a given covariate is used as an independent predictor in one study but as a moderator in another study, that would be obvious to readers and reviewers. Similarly, if measures of the same construct change with each study or if the conditions included in the studies change from study to study, such changes are readily apparent to readers. It is true that some practices are less transparent, such as whether analyses were conducted multiple times as data were collected. In our simulations, therefore, we allowed continued flexibility in this type of practice while constraining those that would be more apparent to readers. If restricting studies to similar sample sizes in addition to the modeled constraints, that would further reduce the ability of p-hacking to produce the sets of consistent results.

### Simulation set-up

To provide a more complete and general view of false findings due to p-hacking in multi-study sets, we undertook a series of simulations (all R simulation code and data can be found at https://osf.io/5kurp/; a ReadMe file in the repository describes how the R files generated and analyzed the data, and summarized the results for each scenario described in the Methods section of this article; the ReadMe file also describes two posted Excel files in which the results are presented in raw or summarized form, respectively).

First, we attempted to replicate the simulations from Simmons and colleagues [4] to ensure that we were examining comparable data dynamics for single studies. Then, we examined how adding additional studies to the set influenced the patterns of results across studies, both when the simulated studies represented the entire set and in the context of potential for selective reporting. Selective reporting is key in that this could be considered an RDF/QRP that has not received much attention alongside other practices. In that respect, we are allowing for more extreme versions of p-hacking associated with a large file drawer than in previous treatments of these matters. However, we are pitting such flexibility against what seemed likely to pose important constraints. That is, we required consistency in methods used across studies (even if the choice of method allowed for considerable flexibility) as well as consistency in the direction of effects. We suspected, and the data bore out, that consistency in methods and effect direction produced relatively low potential for a population null effect to produce a consistent set of false findings. In other words, consistent methods and effect direction protected against the effects of p-hacking. This was especially true as the size of the set of consistent significant studies increases, even when allowing for much selectivity in study reporting. Thus, dire extrapolation from early analyses of false findings in single studies seems relatively unwarranted as yet.

## Methods and results

### Single studies

#### Replication of Simmons et al. (2011)

We began by replicating the simulation of single studies by Simmons and colleagues [4]. Of note, like Simmons and colleagues, we focused on Type I errors in hypothesis testing rather than on effect sizes. This seemed appropriate because the population effect size was zero in all simulations and because many experimental studies focus initially on ruling out a null model as an account of the data before any consideration of effect sizes. Each simulated study had 15,000 replications. All data were generated to have null population effects and normally distributed errors. Simmons and colleagues [4] examined seven situations labeled A, B, C, D, AB, ABC, and ABCD.

In Situation A, a main effect with two conditions was examined for three DVs (*y*_1_, *y*_2_ and *y*_*a*_); *y*_1_ and *y*_2_ were correlated at 0.5 and *y*_*a*_ = (*y*_1_+*y*_2_)2. Each cell size was *n* = 20. The model fit to the data was:

$$y_{i}=b_{0}+b_{1}{condition}_{i}+\epsilon_{i},$$ (1)

where *i* indexed a case in the data set. Situation A involved 3 tests of *b*_1_, one for each DV.

Situation B focused on influences of a two-level independent variable for a single DV (*y*_1_). The same model (Eq 1) was fit to the data and the effect of flexible *n* was examined. Here, if the test of *b*_1_ was not significant for *n* = 20, the cell sizes were increased by 50% to *n* = 30 and *b*_1_ was re-estimated and tested a second time. Thus, Situation B examined the results of 1 + 1 tests of *b*_1_. Situation C involved adding a dichotomous covariate, resulting in two more models fit to *y*_1_.


$$y_{i}=b_{0}+b_{1}{condition}_{i}+b_{2}{covariate}_{i}+\epsilon_{i},$$ (2)

$$y_{i}=b_{0}+b_{1}{condition}_{i}+b_{2}{covariate}_{i}+b_{3}{condition}_{i}*{covariate}_{i}{+\epsilon }_{i},$$ (3)

Taken together, in Situation C, 4 tests (*b*_1_ from Eq 1, *b*_1_ from Eq 2, *b*_1_ from Eq 3, and *b*_3_ from Eq 3) were examined. In Situation D, flexibility in the number of conditions was examined. Instead of two levels, we considered three levels (-1, 0, and 1). By permuting these conditions, the condition variable could take on the values of (-1, 0), (-1, 1), (0, 1), and (-1, 0, 1). Thus, Situation D focused on the 4 tests of *b*_1_ from when Eq 1 was fit to *y*_1_ and the four different ways condition can be permuted.

Situation AB combined situations A and B, resulting in 3+3 tests. The first 3 tests came from the 3 DVs (*y*_1_, *y*_2_, and *y*_*a*_) with *n* = 20 (Situation A) and the next 3 tests came from the same 3 DVs with *n* = 30 if the initial test was non-significant (Situation B). Situation ABC added Situation C onto AB. Because Situation C involved 4 tests after adding a dichotomous covariate, (3×4)+(3×4) or 12+12 tests were examined. Finally, Situation ABCD added Situation D onto ABC. Thus, we had (12×4)+(12×4) or 48+48 tests to examine for Situation ABCD.

In Table 1, our Single Study/Small *n* column independently reproduced the results from Simmons and colleagues [4] for *p* < .05 (middle column of their Table 1, p. 1361). That is, Situations A and B resulted in moderate increases in the probability of Type I Error, with comparatively larger increases based on Situations C and D. As might be expected, combining situations enhanced the probability of Type I Errors, especially by adding Situation C to A and B, and even more so when also incorporating Situation D. Thus, those means of p-hacking that would be most apparent to readers if accumulating evidence across studies (i.e., changing which covariates are included across studies and which conditions are included across studies), especially in combination, tended to lead to the highest probability of Type I errors in single studies.

**Table 1:** Probability of making a Type I error (*p* < .05, two-tailed).

	Single Study		*S* = 2 Studies (Small *n*) Same direction & sig.	
Researcher degrees of freedom (means of p-hacking)	Small *n*	Large *n*	*c* = 1	*c* = 2
Situation A: Two DVs (ρ = .50) and average of DVs	9.54%	9.81%	18.35%	0.31%
Situation B: Adding 50% more observations per cell for first DV	7.66%	7.77%	14.87%	0.29%
Situation C: Controlling for dichotomous covariate or interaction of covariate with treatment	11.45%	11.57%	21.95%	0.37%
Situation D: Dropping (or not) one of three conditions for first DV	12.80%	12.13%	23.86%	0.43%
Combine Situations A and B	14.78%	15.10%	26.81%	0.63%
Combine Situations A, B, and C	31.48%	31.16%	51.32%	1.86%
Combine Situations A, B, C, and D	60.84%	60.11%	79.22%	5.73%

Notes: Type I error is defined as concluding significance with a null population effect. Center columns represent percentages of Type I errors in single studies with small *n* (as in Simmons et al., 2011) and with large *n*. Right columns represent sets of two studies with percentages of samples in which one of two studies was significant and in which both were significant and in the same direction.

Small *n* = 20 per cell + 10 for Situation B (cf. Simmons et al., 2011), and large *n* = 200 per cell + 100 for Situation B.

*S* = Total number of studies, and *c* is the count of significant studies out of *S*.

#### Large *n* replication of Simmons et al. (2011)

In those single-study simulations, each cell size could be *n* = 20 or *n* = 30 (in situations involving Situation B). In the Single Study/Large *n* column of Table 1, we increased *n* 10-fold to examine whether these RDF-induced Type I errors can be reduced by increasing sample size. Large *n* studies have the general benefit of increasing statistical power (e.g., through reducing the standard error of a mean difference of interest). It is interesting to note, however, that increasing *n* did little in the current case to reduce the probability of obtaining a Type I error, regardless of the situation examined. This might not be intuitive in that one might expect larger samples to be more likely to reflect the population null effect. However, because the Type I errors of interest here are not being produced by sampling error (which would be reduced by increasing sample size), these Type I errors are also not reduced by increasing sample size. Thus, at least when the role of sample size in p-hacking is treated in a proportionally equal way across samples (e.g., increasing sample size by 50%), large samples do little to shield one from potential problems associated with p-hacking. We return to this interesting finding in the discussion section of the paper.

### Pairs of studies

#### Choosing one study from the pair

In the 2 Studies columns of Table 1, we examined the probability of observing a Type I error when two studies are conducted. When two studies, each with 3 tests (i.e., Situation A), are conducted, the *c* = 1 column reports the probability of *at least one* test being significant across the two studies. As Nelson and colleagues [22] noted, researchers might conduct more studies than they report. The addition of a second study from which to choose has the anticipated effect. If choosing one study from the two, the probability of a Type I error increases. For the single cases of A, B, C, or D, the probability roughly doubles, though not for the more complex combinations of situations (where the baseline probability of Type I error was already higher). Probability of at least one Type I error would only increase further if 3 or more studies had been conducted as the set from which the researcher is selecting a *single* study to report.

#### Requiring two directionally and methodologically consistent effects

In many cases, however, researchers report all the studies using a particular paradigm. When they do so, they often also use the same methods across studies (e.g., same measures, same covariate[s], same design) and report directionally-consistent effects across studies. To do otherwise would be noticeable to readers and, at least for many of the choices, raise skepticism on the part of readers. Therefore, in our simulations, we examined the probability of Type I errors when the same A, C, D, or combined situations were used in every study and the significant results fell in the same direction across studies (but with retained flexibility in sample size). The *c* = 2 column in Table 1 reports the probability of the same test (i.e., same regression coefficient, same measures, covariates, design) being significant (*p*<.05, two-tailed) with the same direction in both studies. In conditions involving Situation B, the probability of the same test being significant was recorded regardless of whether the original (*n* = 20) or 50% enhanced sample (*n* = 30, when the *n* = 20 situation was not significant) produced the result. We made these assumptions because it would be obvious to readers whether the effect being tested changed (e.g., test of *b*_1_ instead of *b*_3_ in Eq 3; Situation C), measures changed, or conditions included changed across studies, but it would not be as obvious whether additional data were collected. Thus, although important constraints were placed on the test results across studies, these numbers kept the potentially important flexibility in which direction the set of presented studies supported and whether data were or were not added after an initial analysis.

In general, requiring pairs of Type I errors to be consistent in direction and method across two studies dramatically reduced the probability of such pairs of Type I errors compared with requiring only a single study (comparing column *c* = 2 with *c* = 1 in Table 1). That is, even Situation ABCD that would have produced a Type I error over 60% of the time for a single study (Single Study columns of Table 1) or over 79% when selecting a significant study from the pair; column *c* = 1) produces two directionally-consistent Type I errors quite infrequently (less than 6% of the time; column *c* = 2; see Table 1). Thus, requiring that an effect be supported twice using the same methods (measures, covariate treatment, and design) makes it quite difficult for even fairly pronounced p-hacking to produce high levels of directionally-consistent paired Type I errors. This paints a picture that would seem rather different from that painted in previous commentaries extrapolating from the single-study simulations.

### Multiple studies

Of course, Nelson and colleagues [22] might have had sets of studies in mind with more than two studies, and they might expect some level of selective reporting to occur in those sets. Even with such selection, however, readers can diagnose certain means of p-hacking. For example, the reader generally knows whether a given construct was measured the same way across studies or was measured with a different set of items in each study. Similarly, the reader can tell whether different studies use different covariates (or any covariates are treated differently across studies) or whether the design of the study (i.e., which conditions are included) remains constant across studies or not. Many sets of studies present a consistent set of measures, covariates, and designs as well as a directionally-consistent set of findings. Would such result patterns be easy to produce by finding the “right set” of practices when population effects are null? If the influence of such constraints in the 2-study set are any indication, perhaps even fairly extreme versions of p-hacking would not have a particularly easy time making *anything* appear significant once consistent methods and direction of effects are required across studies. If so, then one would not expect large proportions of studies (e.g., 50% or more) to provide directionally-consistent significant results. At the same time, especially with relatively substantial p-hacking, one would expect at least some significant results to occur somewhere in the set, especially as the size of the set grows. We examined such questions first using a simulation of 10-study sets (where we document the likelihood of significant results from 0–10 of the studies given directional and methodological constraints). Then, we examined the likelihood of different-sized sets of directionally- and methodologically-consistent significant studies from a set of up to 50 total studies. In each case, we expected the constraint of results being directionally-consistent and using the same methods (for 2 or more studies) to result in relatively low likelihood of consistent sets of Type I errors.

#### 10-study sets

Suppose that the researcher had the resources to collect data for 10 studies. How often would Type I errors occur for the same test (i.e., same regression coefficient, measures, covariates, and design) with the same directional effects across a set of *c* such studies out of the *S* = 10? Table 2 presents these results (maintaining flexibility in whether additional data are collected after an initial analysis). Consider Situation ABCD. Because of the large number of tests (48+48), there is only 0.01% chance of having no Type I errors. With 10 studies and multiple tests, 14.31% of the time Type I errors occurred in only 1 of the 10 studies. The most likely outcome, occurring 62.55% of the time was 2 directionally-consistent Type I errors out of the 10 studies. Thus, it would be fairly frequent for 10 studies of population null effects (with fairly heavy p-hacking) to produce 2 studies with the same significant test result in the same direction. About one fifth of the time, 21.05%, 3 directionally-consistent studies were produced by the 10 studies. Having the same significant result in the same direction occur for 4 studies out of 10 is much less likely (1.93% of the time), and even less for 5 or more.

**Table 2:** Likelihood of making a Type I error out of *S* = 10 studies.

	Highest *c* number of studies (out of 10) with a significant result from the same test with same direction										
Researcher degrees of freedom	0	1	2	3	4	5	6	7	8	9	10
Situation A: Two DVs (ρ = .50) and average of DVs	35.45%	53.43%	10.35%	0.74%	0.03%	0.00%	0.00%	0.00%	0.00%	0.00%	0.00%
Situation B: Adding 50% more observations per cell for first DV	44.60%	44.57%	9.71%	1.07%	0.05%	0.00%	0.00%	0.00%	0.00%	0.00%	0.00%
Situation C: Controlling for gender or interaction of gender with treatment	28.03%	58.57%	12.46%	0.91%	0.03%	0.00%	0.00%	0.00%	0.00%	0.00%	0.00%
Situation D: Dropping (or not) one of three conditions for first DV	26.59%	59.59%	12.91%	0.85%	0.05%	0.00%	0.00%	0.00%	0.00%	0.00%	0.00%
Combine Situations A and B	19.90%	57.15%	20.07%	2.69%	0.18%	0.01%	0.00%	0.00%	0.00%	0.00%	0.00%
Combine Situations A, B, and C	2.25%	46.63%	42.64%	7.89%	0.57%	0.02%	0.01%	0.00%	0.00%	0.00%	0.00%
Combine Situations A, B, C, and D	0.01%	14.31%	62.55%	21.05%	1.93%	0.13%	0.01%	0.00%	0.00%	0.00%	0.00%

*Note*. The probabilities in the cells sum up to 100% across the rows.

There might be some different ways to interpret the meaning of such results. On one hand, when using fairly heavy-handed p-hacking (Situations ABC or ABCD), a large percentage of the time, 2 or 3 directionally-consistent results are produced out of the 10 studies (about 50% of the time for Situation ABC, and 83% of the time for Situation ABCD–both heavily weighted toward finding 2 rather than 3 directionally- and methodologically-consistent results). Two or 3 directionally-consistent significant results out of 10 studies would be highly unlikely when dealing with population null effects with no p-hacking. Yet, the introduction of p-hacking clearly makes even directionally-consistent results (in pairs or 3s) more likely. Even so, it is still quite infrequent to produce 4 or more directionally-consistent results out of the 10 (even with Situation ABCD, and especially with anything less extreme). If one needed, say, a minimum of 3 directionally-consistent significant results to make a compelling article, conducting 10 studies of a population null effect and hoping that p-hacking will produce such a set would not be a particularly successful strategy. That is, even after conducting the 10 studies, only a little over 20% of the time would one have 3 or more directionally-consistent significant results to report in an article, even using Situation ABCD (and a lot less for any of the less extreme versions of p-hacking).

The final row of Table 2 as well as the columns corresponding to 3 or more significant effects provide some important points to consider. In the 10-study setting, once a researcher reports 3 directionally-consistent significant results using the same measures, covariates, and design, one can probably infer that no mild form of p-hacking is responsible for the consistent results even with severe non-reporting. That is, the single practices (A, B, C, or D) are not at all effective in producing sizeable sets of consistent significant tests. The combination of flexible measures and flexible sample sizes (Situation AB) does little to increase the probability of sets of directionally-consistent significant tests. Even the ABC combination barely gets above the nominal 5% rate. The only way to move far beyond this rate is to move to the ABCD combination (where nobody is undertaking such practices alongside severe non-reporting by accident). But even here, such extreme practices fail to produce 3 or more directionally-consistent significant effects far more often than they succeed. Also, to reach 3 or more directionally-consistent significant effects and to have any chance at all (and still well below the 61% observed for a single study), one must also assume that the file drawer is at least twice the size of the reported set. Even if all of this is true, the odds are still tilted distinctly against finding the 3 directionally-consistent significant tests with a null population effect. Needing a file drawer twice the size of one’s reported study set (or larger) is not a fast track to success. Depending on the logistical challenges of one’s paradigm, it might not even be feasible. Considering programs of research, obtaining a second set that falls in the same direction as well will even be that much less likely. So far as that goes, another researcher attempting to match the original set will be similarly unlikely even if both researchers are engaging in extreme p-hacking and extreme non-reporting. Thus, when one sees separate sets of studies (within or across researchers) that use consistent methods and find directionally-consistent significant results, it becomes difficult to account for such outcomes with reference to p-hacking.

#### Up to 50-study sets

**Fig 1:**
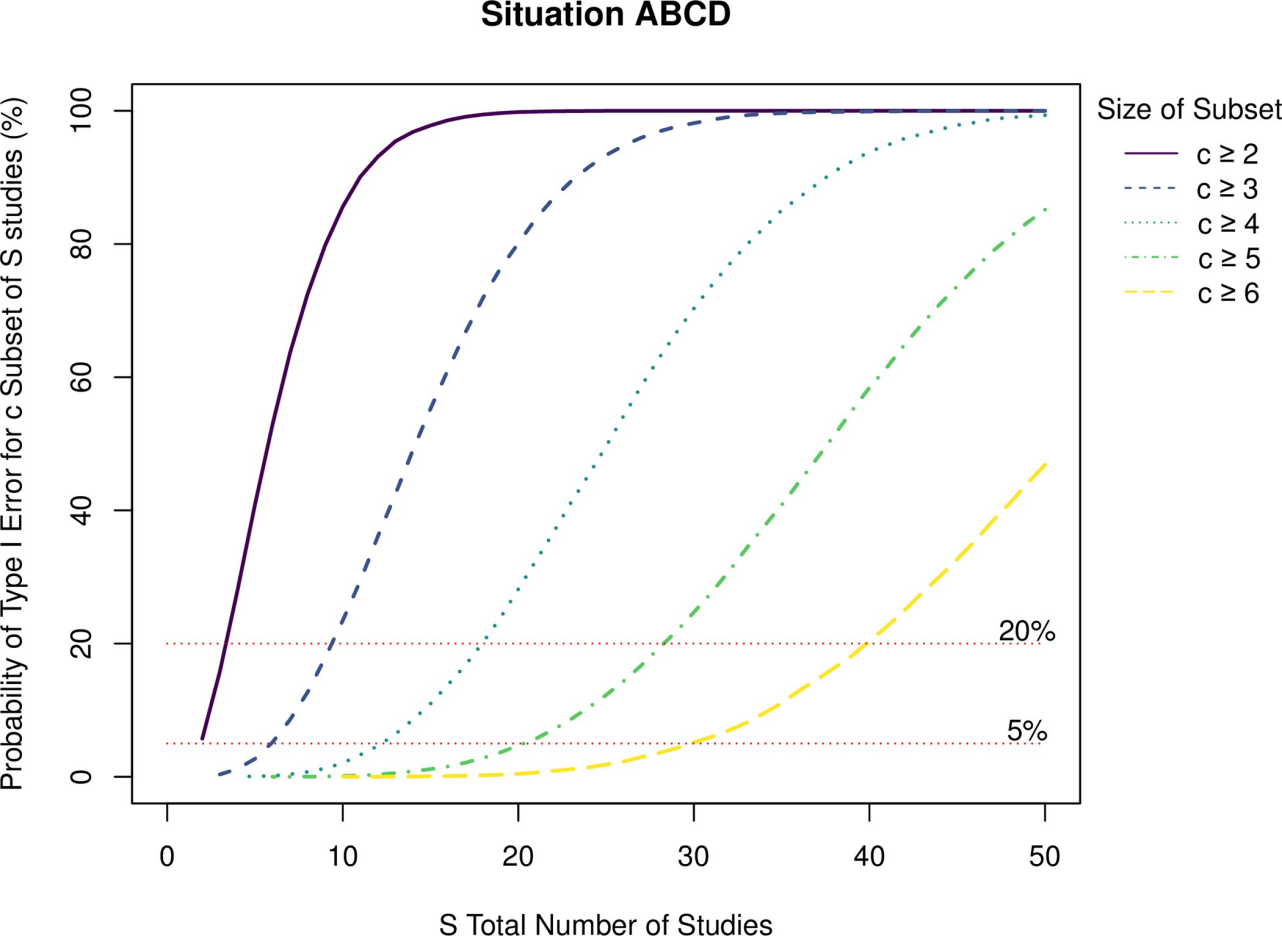
Probabilities of c or more directionally-consistent Type I errors for situation ABCD.

The online supplement at https://osf.io/5kurp/ includes a table (S1) presenting the probability of obtaining *c* or more directionally-consistent results (where *c* ranges from 2 to 6) out of S studies (where *S* ranges from *c* to 50). Fig 1 summarizes the probability of *c* or more directionally-consistent results for Situation ABCD (the combination producing the highest probability of Type I errors in the single-study simulations presented by Simmons and colleagues [4]). Each point on a given line represents the probability of obtaining *c* or more directionally-consistent results using the same measures, covariates, and design across studies while allowing for different sample sizes (i.e., either 20 per cell or, if not significant, retesting with 30 per cell) for the number of total studies (*S*) listed on the horizontal axis. As points of reference, there are faint horizontal reference lines at 5% and 20% probability of obtaining that set of directionally-consistent significant results.

We extended to this case, in part, to examine how much selectivity in study reporting would be necessary for a given number of directionally- and methodologically-consistent significant results to be obtained. Such information would provide important context for the plausibility of p-hacking-plus-selective-reporting to produce the types of study sets of interest. We expected that the larger the set of consistent significant studies (i.e., the larger the *c* value), the larger the file drawer of non-significant (and likely directionally inconsistent) studies must be to provide any sizeable chance of producing the set of *c* directionally- and methodologically-consistent significant studies. The real amount of selectivity in study reporting might often be rather invisible to readers, except in the case where authors report that the manuscript includes all studies using a given paradigm or design. Even so, the feasibility of a selective-reporting-plus-p-hacking account of the data might be assessed to some degree based on such simulations.

Recall that Fig 1 refers to Situation ABCD (i.e., the combination of practices that led to the largest proportion of Type I errors– 61%–in our replication of the previous simulations [4]). One set of potential observations of interest in Fig 1 (expanded in Table S1 in the online supplement) concerns the points at which the overall chance of obtaining the set of significant results exceeds 5% (a Type I error rate that people generally accept for any given single study that they analyze). When considered as study sets, the probability of the set representing Type I errors is not less than 5% at all for *c* ≥ 2 studies (i.e., a pair of directionally-consistent significant studies from a set of *S* = 2 or more studies). However, for Situation ABCD, the probability of Type I error creating the set is below 5% for *c* ≥ 3 if *S* = 5, for *c* ≥ 4 if *S* = 12, for *c* ≥ 5 if *S* = 20, and for c ≥ 6 if *S* = 29. Thus, even setting aside whether relying on p-hacking to produce significant results would be an “effective strategy,” the overall Type I error rate remains in a range with which people are generally comfortable for a single study when presenting 3 directionally-consistent significant studies (with the same measures, covariate use, and design) with no more than 5 total studies, 4 directionally-consistent significant studies with no more than 12 total studies, 5 directionally-consistent significant studies with no more than 20 total studies, or 6 directionally-consistent significant studies with no more than 29 total studies.

In terms of whether people could produce a body of work through egregious p-hacking, perhaps another relevant referent might be the point at which the rate of Type I errors producing the pattern reaches 20%. A “hit rate” of 20% *after conducting multiple studies* would seem like a rather poor strategy in that it would require a lot of data collection for still probabilistically unlikely returns. Nonetheless, a 20% “hit rate” is reached or exceeded in Situation ABCD for *c* ≥ 2 directionally-consistent results when *S* = 4, for *c* ≥ 3 directionally-consistent results when *S* = 10, for *c* ≥ 4 directionally-consistent results when *S* = 18, for *c* ≥ 5 directionally-consistent results when *S* = 29, and for *c* ≥ 6 directionally-consistent results when *S* = 40. In other words, to reach a 20% hit rate for producing 2 directionally-consistent studies, one’s file drawer would have to be twice the size of the set of significant results to have even a modest chance of success. At 4 studies, one’s file drawer would have to be more than three-times the significant set size, and at 6 studies, one’s file drawer would have to be more than five-times the size of the presented set of significant studies. And this is just to be able to reach a hit rate of 20% (i.e., failing 4 times out of 5)! Similar to the 10-study sets results discussed earlier, it would seem like a losing effort to conduct 10, 18, or even more studies just to have a 20% chance of producing 3, 4, or more directionally-consistent results based on p-hacking. This is especially true given that the reported results might not then be consistent with sets of significant studies produced by later data collections. It would be much more productive to study effects with non-zero values in the population!

## Discussion

Previous work on p-hacking of single studies suggested that significant statistical tests can occur with troubling regularity even when effects are null in the population [4]. Consistent with such notions, the current simulations suggest that a *single study* is clearly vulnerable to p-hacking, especially if a researcher is willing to engage in a fairly extreme version of p-hacking and is willing to engage in post hoc interpretation of whatever results are identified in such analyses. Mild versions of p-hacking do not produce nearly the same level of vulnerability for single studies, but extreme versions certainly can, especially if there is any level of selective reporting in choosing the study from a larger set. In such settings, a researcher would also have to be willing to either suppress later studies that happen to produce opposite results or engage in further ad hoc interpretation of study differences (that would be unlikely to withstand direct studies of proposed moderators aimed at explaining the differences across studies).

### Sample size versus replication-based protections

One new and important aspect of the current simulation results is to note that single studies do not gain any real protection by increasing sample size, at least not for the versions of p-hacking we examined across the different sample sizes. This makes sense because the influences of p-hacking are not due to sampling variability (which would be reduced by increased sample size). Intuitively, one might have imagined that larger samples would simply be more likely to reflect the “truth” of the null effect in the population data and that reduced sampling variability would also hedge against many or all forms of p-hacking. Yet, large-sample studies remain just as vulnerable to p-hacking as small-sample studies for the implementations that we and others have examined. This does not necessarily mean that every form of p-hacking would have equal influences across sample sizes, however. For example, though we treated addition of data before re-analysis as adding a given proportion of the current sample size (i.e., analyze, add 50% more data, then analyze again), one could just as well have defined the practice not in proportional terms. For example, one could have viewed an increase in sample size from 20 per condition to 30 per condition not as increasing cell sizes by 50% but, instead, as increasing sample size by 10 participants. Though we did not examine this in our simulations, we would expect that adding 10 participants would have greater potential for increasing the Type I error rate when adding to a sample of 20 per cell rather than a sample of 200 or 2000 per cell. When instead equating the proportion of increase in sample size across analyses, we found similar rates of increase in Type I error across initial samples of 20 per cell or 200 per cell. We suspect that future simulations with baseline samples of 200 per cell would also parallel the obtained results with baseline samples of 20 per cell when examining larger study sets (as in Fig 1 and Table S1), as long as the proportional increase in sample size is again equated across the 20–30 and 200–300 scenarios for Situation B. It is important to realize, however, that large samples provide relatively little protection against many forms of p-hacking and considerably less so than replications (even with selective study reporting) of directionally- and methodologically-consistent results.

### Multiple-study sets

Perhaps the most important conclusion to take from the current simulations is that they provide important qualifications to any extrapolation from single studies to multi-study sets regarding the influence of p-hacking. It might be a natural assumption that high single-study false finding rates would indicate great ease in bringing about multi-study sets of consistent results. However, simulations that directly incorporate consistency of methods (i.e., measures, covariate treatment, and design) across studies lead to markedly different conclusions. When one moves to the world of 2 or more studies, it becomes difficult (nearing impossible) to produce sets of directionally-consistent Type I errors with mild or benign forms of p-hacking that a researcher might undertake unwittingly or somewhat innocently. For example, there has been much attention to inflated Type I errors created by testing for significance and then adding additional participants if not significant. Yet, at least given the parameters of the present simulations, when reporting the only 2 studies conducted, none of the practices in isolation produced much in the way of consistent Type I errors across the studies (Table 1, right column). Extreme non-reporting could increase the probability of a pair of consistent significant results, but with clear limits. For example, in Table S1, even if conducting *S* = 6 studies (i.e., withholding twice the number of studies as are reported), for any of the individual p-hacking practices, the probability of *c* ≥ 2 directionally-consistent Type I errors was less than 5.73%. Given that the population value of the effect in these simulations is zero, it also seems likely that a sizeable proportion of observed effects with consistent methods across studies would fall on the opposite side of the distribution. Thus, researchers should have a good idea that the evidence is not strong or consistent for an effect that actually represents a pair of Type I errors in such settings. More generally, it seems that producing sets of consistent p-hacked Type I errors heavily relies on both extreme p-hacking and on extreme non-reporting (e.g., reporting *c* ≥ 4 directionally consistent results after conducting *S* = 17 studies, omitting 13 non-significant and/or directionally-inconsistent results for Situation ABCD). Indeed, all of the single practices require very high levels of non-reporting, and even the AB combination does not produce many sets of Type I errors without pretty notable non-reporting (see S1 Table).

### Practical implications

#### Evaluating study sets

The bottom-line practical question at hand is, when a researcher reports a set of studies, how plausible is it that the reported set could represent a set of Type I errors resulting from p-hacking? Previously, researchers have been left largely to take single-study estimates and extrapolate to what they imagine might happen for larger study sets. In so doing, it seems that some have accorded borderline magical properties to p-hacking, imagining that it would be easy to produce large study sets of seemingly consistent results based on p-hacking practices alone. On some level, this is understandable. The original Simmons et al. article [4] and our own simulations showed that, when the reported study set is 1, extreme p-hacking has a reasonably good chance of producing a significant effect, and one need not assume the existence of a file drawer for this to occur. In short, a single study result can be plausibly attributed to p-hacking if one assumes relatively severe versions of the various practices, especially in combination. More modest versions of the same practices are only moderately successful at producing a Type I error even in the 1-study case, but one might suspect more extreme versions of p-hacking if, for example, a covariate is being treated differently than in related literature or some other practice seems out of the ordinary. In such cases, practices like pre-registration [27, 28] might be important to assure readers that the results are not due to various forms of p-hacking. However, as our simulations suggest, a methodologically-consistent study set is more robust against p-hacked Type I errors, especially as the size of the set increases.

In contrast, consider an article that presented a set of 3 studies with a consistent effect and set of methods. Our simulations suggest that, even with rather severe p-hacking (i.e., Situation ABCD), the file drawer would generally have to be 6–7 studies to have any reasonable chance of producing the 3 studies, and even then, it is not all that likely. A reader might then reasonably infer that, if this is a false finding, it was not produced very innocently. Undertaking every possible combination of four p-hacking tactics and then concealing two thirds of one’s studies does not happen by accident and without awareness, and it seems quite unlikely to represent innocently “fooling oneself.” The milder forms of p-hacking (e.g., one practice on its own) would be highly unlikely to have produced these three studies because the file drawer would get much larger, and that extreme level of non-reporting does not happen without awareness of what one is doing (or without being aware that many of the withheld results go in the opposite direction of those being reported).

Given a total set of 9–10 studies, one might consider the methodological parameters of the reported studies (e.g., nature of the research paradigm, setting, population sampled, sample size, design complexity) and assess the likely time/resource requirements for amassing the study set. Given these parameters, one could estimate the likely time and expense it would have taken to amass a set of 10 studies. If it would take 10 years or many thousands of dollars, it might not seem plausible that a researcher would take that long or spend that much to have a 20% chance of producing a set of studies that might be published in one article. In contrast, if it would take much less time or fewer funds to produce that set of studies, perhaps it would seem believable. Logistics would seem central for estimating what a plausible file drawer could be, even if a person were assumed to be p-hacking freely. One could also observe that the constraints are only more stringent if the researcher is p-hacking to match a previously demonstrated effect, especially one based on a particular set of methodological choices.

#### Overall (lack of) success of p-hacking

With more selective reporting (i.e., a larger file drawer), perhaps the strategy would become a bit more capable of producing consistent sets of studies. At 6 total studies (i.e., 4 in the file drawer), the chance of getting 2 or more directionally-consistent studies to be significant is approximately 53% for Situation ABCD. Even here, this 50–50 shot is only true if engaging in a rather severe form of p-hacking. More mild versions of p-hacking would require larger file drawers (i.e., more than twice the number of non-significant studies as significant ones), and this becomes even more the case as the set size of directionally-consistent significant studies increases. That is, to reach or exceed a 50–50 chance of producing 3 or more directionally-consistent studies, it would take more than 50 total studies for Situation A, would require 48 total studies for Situation B, and would require 47 total studies for Situations C or D (see Table S1). When combining types of p-hacking, Situation AB would still require 34 total studies to exceed a 50–50 shot at producing 3 directionally-consistent significant studies, and Situation ABC would still require 22 total studies (see Table S1). None of these seem like viable strategies for a productive research career.

#### Deliberate rather than accidental

When publishing (and evaluating) study sets, to make p-hacking a plausible explanation for those studies would require rather extreme practices, a rather small set of significant studies, and assumption of substantial file drawer of unreported studies. Thus, on some level, a p-hacking explanation requires assuming that researchers p-hack in a rather cynical and deliberate way. To be clear, we acknowledge that some practices related to p-hacking might be fairly subtle, and researchers could sometimes be convinced that the practice is justified in the particular case they are facing. However, to undertake the fuller set of practices needed to reach high probabilities of Type I errors, it seems more likely that researchers would be aware of an intentional exploration of rather arbitrary analysis alternatives. In such a situation and given that such deliberate uses still produce somewhat low probabilities of producing sets of significant studies, one might ask why such a person would bother with p-hacking rather than making up their data. We think it highly unlikely that lots of researchers are cynical or amoral enough to be undertaking blatant fraud, and we also think it highly unlikely that lots of study sets (especially the large ones that appear in many articles in top journals) represent sets of Type I errors produced through p-hacking. Even if one thought such practices were more typical, if undertaken without faking data, one would also have to acknowledge that such practices are not very practical, because the logistical challenges would often be sizeable and the likelihood of success quite low. As demonstrated in the current simulations, the only way around this practicality issue would be to allow different practices across studies, and these would likely involve recognizable signs of p-hacking, such as different sets of measures, different treatment of covariates, or different substantive conditions across studies. As the simulations clearly show, however, sets of directionally-consistent Type I errors become highly unlikely when the study sets use consistent methods (e.g., the same measures, design, analyses, covariates, etc.) across studies and as the set size grows.

### Directions for future research

Our simulations emulate multiple studies that are direct replications of one another. Yet, to enhance various forms of validity [29], studies reported in a single article often operationalize independent variables differently, examine different populations, or utilize different measures, for example. In fact, the typical profile of a multi-study paper is probably an initial demonstration of a key effect in a first study and then various extensions of the initial study or moderation of the key effect. Thus, one might have a set of conditions that are replicated across the studies but also other effects (extensions or moderation) that are demonstrated only once or perhaps 2–3 times. If so, many sets of studies would have some effects that (presuming methodological consistency) are likely to be rather robust to p-hacking alongside other effects that might be less robust. However, being locked into consistency across demonstrations of the key effect (e.g., measures used for key predictors or dependent measures, covariates treated similarly, etc.) would also constrain many of the options for p-hacking of the new extensions or moderation effects. After all, any p-hacking undertaken in search of a new effect cannot alter how the key effect is demonstrated across studies. In addition, the direction of the results for any new extensions or moderation would have to fit in a logical way with the explanation used to account for the key effect. We look forward to future simulations that address this more general scenario. We suspect that the constraints in one part of a study set (e.g., for a key replicated effect) would also make it unlikely to produce sets of independent extensions or moderation effects that are conceptually coherent. Even so, it is possible that any given publication includes some effects that are relatively unlikely to have been produced by p-hacking and other effects that might remain potentially vulnerable to such an explanation.

### Conclusions

The point of this article was not to argue that p-hacking is acceptable or has no consequences. However, to assume that p-hacking is equally plausible as an account of the data across all sets of studies is also clearly incorrect. P-hacking is not magical, and p-hacking is unlikely to account for large sets of directionally-consistent significant results, especially when methods used across the studies are consistent and when there is little selectivity in terms of which studies are presented in a given article. Despite calls for single large *n* studies in place of multiple smaller *n* studies [30, 31], our simulations indicate that multiple smaller *n* studies might be substantially more protective against Type I errors due to the types of practices we examined (assuming that flexibilities associated with adding data are proportionally the same across large *n* and small n studies). Attempts to attribute sets of study results to p-hacking make a variety of (often unstated) assumptions, many of which might be questionable given the methods used in the reported studies. Like a number of other methodological contexts, a crucial question might regard the overall number of studies conducted using a given paradigm, because the amount of selectivity required for p-hacking to produce consistent sets of results is often quite large. Our hope is that one impact of the current discussion might be to encourage researchers to pay greater attention to the set of research results across studies rather than only (or predominantly) focusing on results of individual studies within the set. Paying greater attention to patterns of evidence across studies could reduce motivations to seek “perfect” evidence in each study (and potentially withhold the imperfect evidence). The result would likely be to enhance the accuracy of claims made about study sets [13, 16].
